# The Relationships between Intestinal Permeability and Target Antibodies for a Spectrum of Autoimmune Diseases

**DOI:** 10.3390/ijms242216352

**Published:** 2023-11-15

**Authors:** Datis Kharrazian, Martha Herbert, Jama Lambert

**Affiliations:** 1 Harvard Medical School, Boston, MA 02215, USA; drmarthaherbert@gmail.com; 2 Massachusetts General Hospital, Charlestown, MA 02114, USA; 3 Department of Preventive Medicine, Loma Linda University School of Medicine, Loma Linda, CA 92354, USA; 4 Higher Synthesis Foundation, Cambridge, MA 02138, USA; 5 Independent Researcher, Puerto Vallarta 48300, Jalisco, Mexico; jamalambert@gmail.com

**Keywords:** intestinal permeability, zonulin, autoimmunity, zonulin/occludin antibodies, leaky gut

## Abstract

The worldwide prevalence of autoimmune diseases that have limited treatment options and preventive strategies is rapidly rising. There is growing evidence that the microbiota and the integrity of the intestinal barrier play a role in autoimmune diseases. The potential to evaluate intestinal barrier integrity for susceptible individuals and to determine whether restoring intestinal junction integrity impacts autoimmune diseases is an important area of research that requires further attention. In the intestinal permeability model of autoimmune diseases, the breakdown of the intestinal tight junction proteins (zonulin/occludin) allows bacteria, toxins, undigested dietary proteins, and other antigens to pass into the lumen, thereby increasing the number of inflammatory reactions and the activation of immune cells throughout the body. In this study, we investigate the relationship between zonulin/occludin antibodies, which are used to determine intestinal permeability, with autoantibodies used to diagnose autoimmunity. Our investigation may identify significant levels of circulating autoantibodies in human subjects with intestinal permeability compared to those without intestinal permeability. Furthermore, we identified that significant positive linear correlations between serum occludin/zonulin antibodies and circulating autoantibodies could be used to determine autoimmune diseases.

## 1. Introduction

The worldwide incidence and prevalence of virtually all autoimmune diseases has risen steadily over the past 30 years [1]. There is growing evidence that the imbalances in the gut microbiota and impaired integrity of intestinal tight junctions may play a role in the development of autoimmune disease [2,3]. In the intestinal permeability model of autoimmune disease, the breakdown of the intestinal tight junctions allows bacteria, toxins, undigested dietary proteins, and other antigens to pass into the lumen, thereby increasing inflammatory reactions within the gastrointestinal environment and throughout the body [4,5]. The loss of proper macromolecule trafficking can induce immune dysregulation, impair tolerance, and lead to several mechanisms that set the stage for the expression of autoimmune diseases in susceptible individuals [6,7].

**Figure 1:**
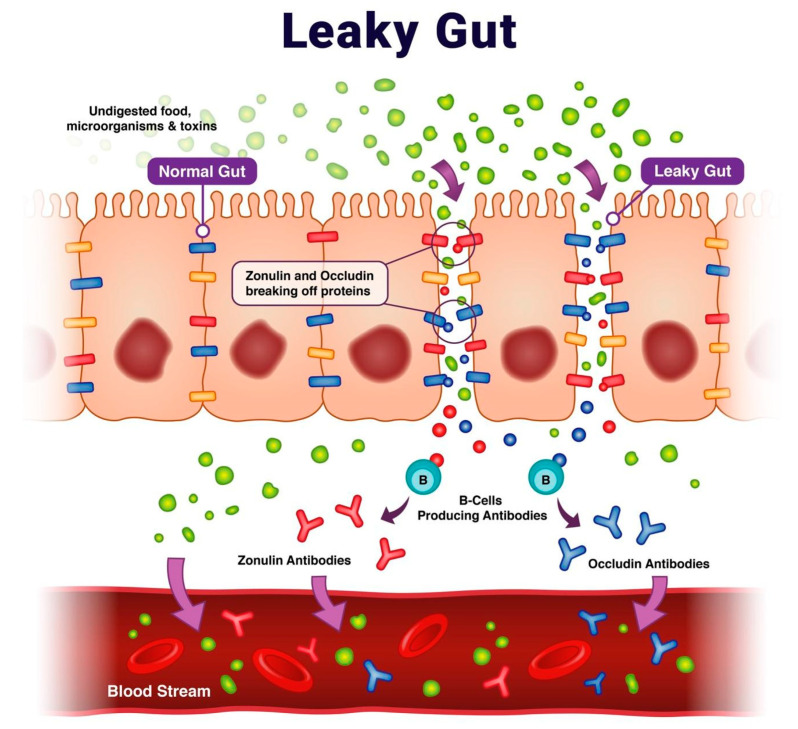
Intestinal permeability referred to as “leaky gut” pathophysiology and the formation of occludin/zonulin antibodies.

The intestinal epithelium maintains its impermeability from large undigested protein macromolecules and various pathogens with occludin junctional adhesion molecules. These tight junction proteins are regulated by zonulin. Intestinal cells synthesize zonulin, which is used to reversibly regulate intestinal permeability [8]. Serum antibodies against intestinal tight junction proteins, zonulin, and occludin develop with intestinal permeability and are found to be reliable, stable, and reproducible biomarkers for identifying intestinal permeability (Figure 1) [9,10].

In this study, our objective was to assess the relationship of autoantibody markers of autoimmunity with intestinal permeability as clinically determined by the presence of elevated zonulin/occludin antibodies. This includes comparing mean autoantibodies in human subjects with and without intestinal permeability and comparing autoimmunity risk for human subjects with and without intestinal permeability.

## 2. Results

**Figure 2:**
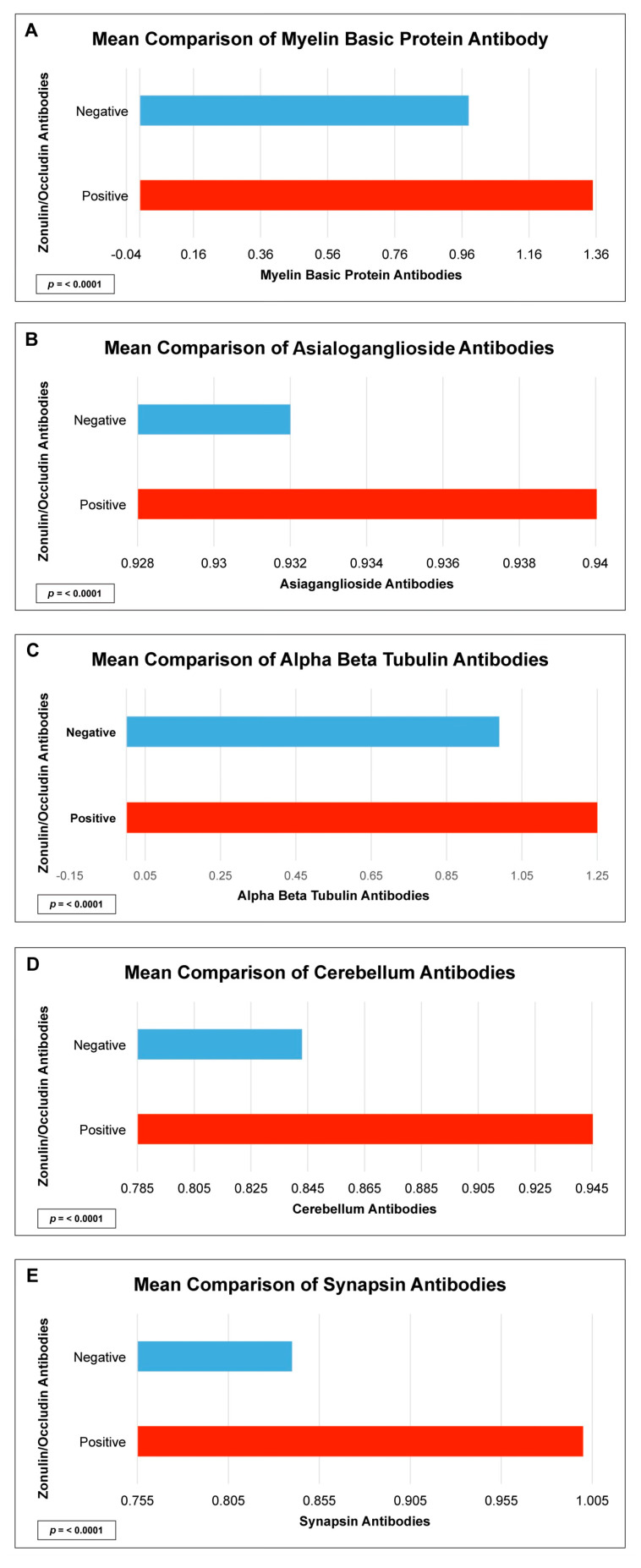
Comparison of subjects with positive and negative serum zonulin/occludin antibody levels and neurological tissue antibodies; (**A**) myelin basic protein, (**B**) asialoganglioside, (**C**) alpha/beta tubulin, (**D**) cerebellum, and (**E**) synapsin. Positive zonulin/occludin antibodies were defined as levels greater than two (2) standard deviations from the mean. The *p*-value for all comparisons were <0.0001.

**Figure 3:**
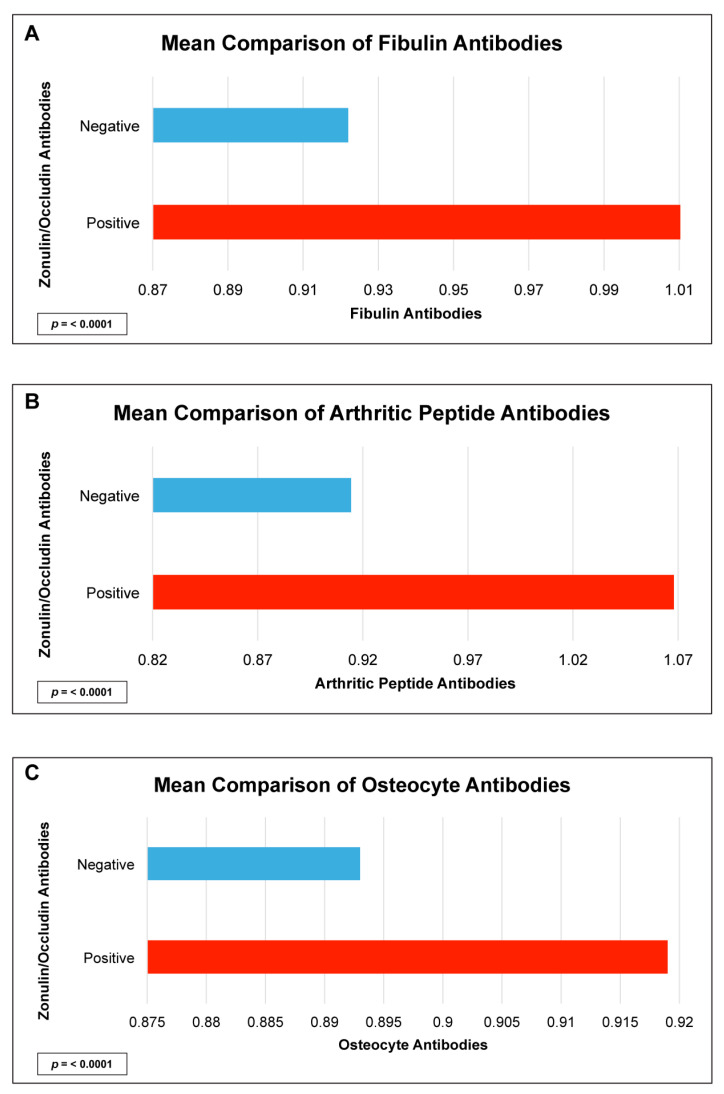
Comparison of subjects with positive and negative zonulin/occludin antibody levels and various joint tissue antibodies: (**A**) fibulin, (**B**) arthritic peptide, and (**C**) osteocyte. Positive zonulin/occludin antibodies were defined as levels greater than two (2) standard deviations from the mean. The *p*-values for all comparisons were <0.0001.

**Figure 4:**
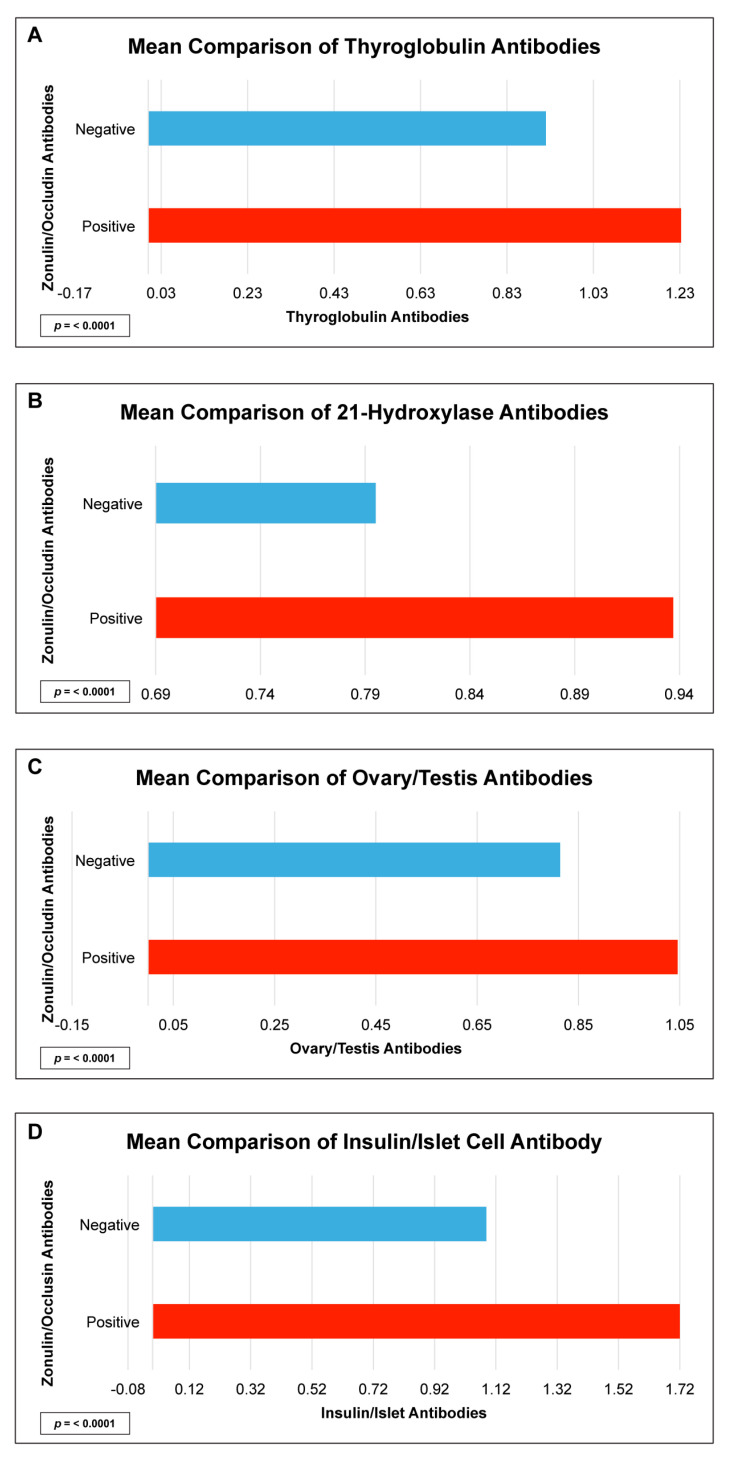
Comparison of subjects with positive and negative zonulin/occludin antibody levels and endocrine tissue antibodies: (**A**) thyroglobulin, (**B**) 21-hydroxylase, (**C**) ovary/testis, and (**D**) insulin/islet cell. Positive zonulin/occludin antibodies were defined as levels greater than two (2) standard deviations from the mean. The *p*-values for all comparisons were <0.0001.

**Figure 5:**
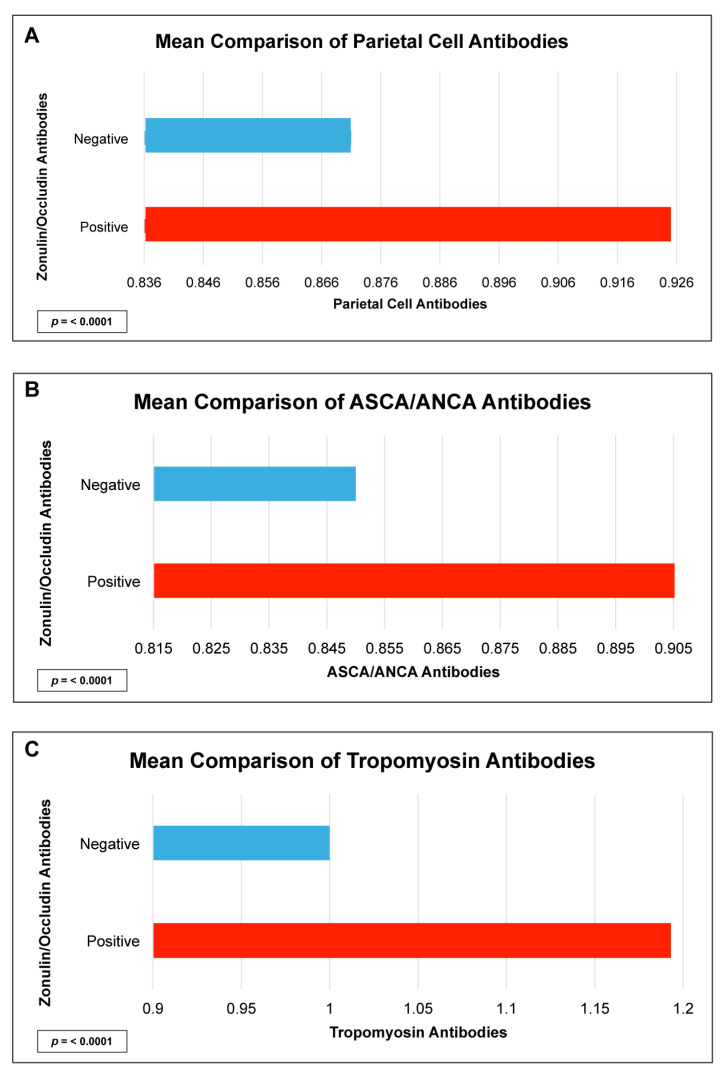
Comparison of subjects with positive and negative zonulin/occludin antibody levels and intestinal tissue antibodies: (**A**) parietal cell, (**B**) ASCA/ANCA, and (**C**) tropomyosin. Positive zonulin/occludin antibodies were defined as levels greater than two (2) standard deviations from the mean. The *p*-values for all comparisons were <0.0001.

**Figure 6:**
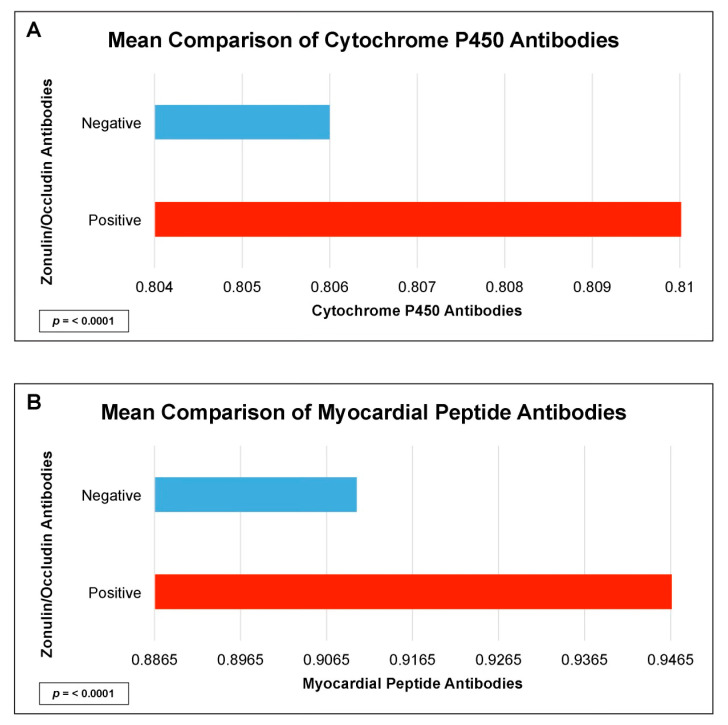
Comparison of subjects with positive and negative zonulin/occludin antibody levels and tissue antibodies: (**A**) myocardial peptide, and (**B**) cytochrome P450. Positive zonulin/occludin antibodies were defined as levels greater than two (2) standard deviations from the mean. The *p*-values for all comparisons were <0.0001.

**Figure 7:**
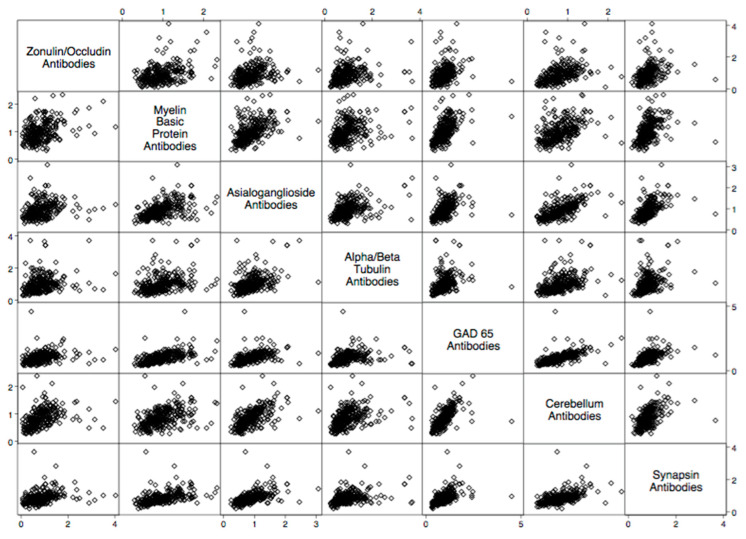
Scatter matrix of positive linear relationships with neurological tissue antibodies and zonulin/occludin antibodies.

**Figure 8:**
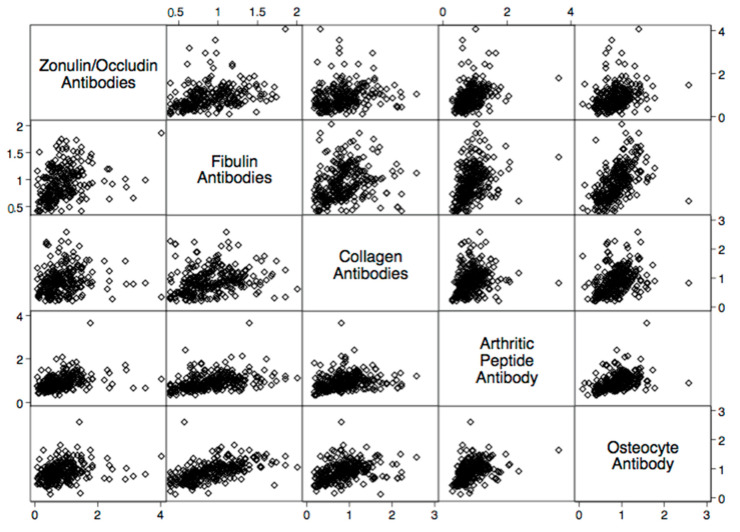
Scatter matrix of positive linear relationships with joint tissue antibodies and zonulin/occludin antibodies.

**Figure 9:**
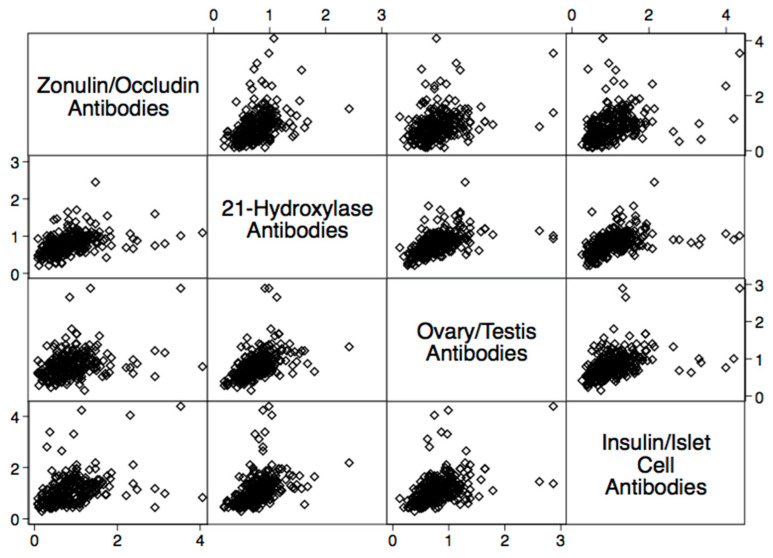
Scatter matrix of positive linear relationships with endocrine tissue antibodies and zonulin/occludin antibodies.

**Figure 10:**
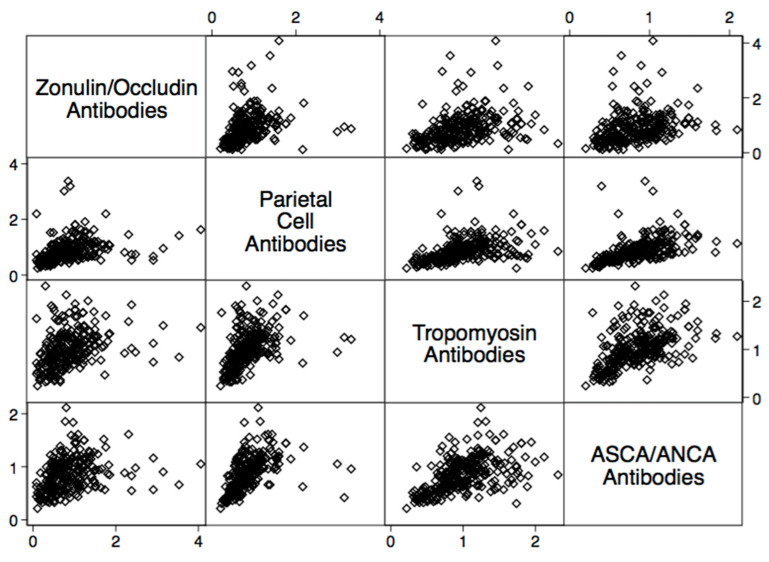
Scatter matrix of positive linear relationships with gastrointestinal tissue antibodies and zonulin/occludin antibodies.

**Figure 11:**
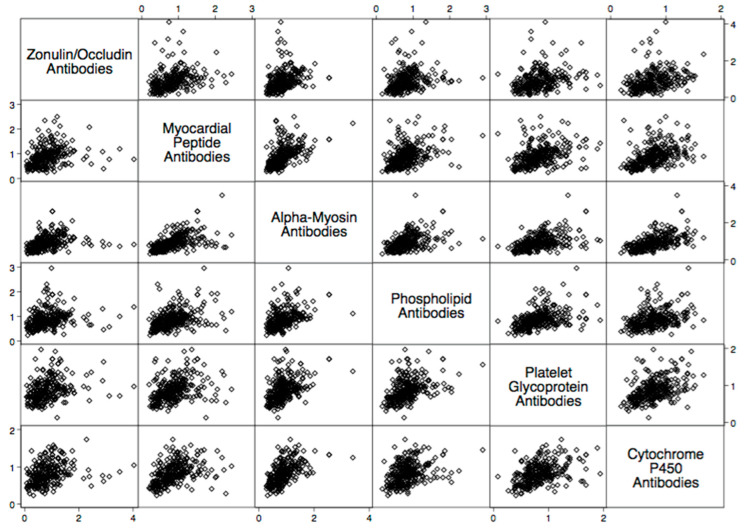
Scatter matrix of positive linear relationships with tissue antibodies throughout the body and zonulin/occludin antibodies.

There were statistically significant elevations in mean autoimmune antibodies in subjects with intestinal permeability (zonulin/occludin positive) as compared to subjects without intestinal permeability (zonulin/occludin negative) for 17 out of 24 autoimmune target proteins (Figure 2, Figure 3, Figure 4, Figure 5 and Figure 6). With logistic regression analyses, there was a 3- to 30-fold increase in the odds of detecting elevated autoimmune target protein antibodies in subjects with intestinal permeability compared to the odds of developing autoimmune target proteins in subjects without intestinal permeability (Table 1). There were also statistically significant positive linear correlations with zonulin/occludin antibodies and autoimmune target protein antibodies (Figure 7, Figure 8, Figure 9, Figure 10 and Figure 11). The correlations coefficients were small to moderate (Table 2).

## 3. Discussion

Our investigation may identify significant levels of circulating autoantibodies in human subjects with intestinal permeability compared to those without intestinal permeability. Furthermore, we identified that significant positive linear correlations between serum occludin/zonulin antibodies and circulating autoantibodies could be used to determine autoimmune diseases. An important finding of our study indicates that intestinal permeability has a generalized role in autoimmune diseases, involving the brain, the endocrine gland, joints, smooth muscles, the cardiovascular system, etc. These findings support the notion that autoimmune diseases throughout the body may share a centralized role, involving the integrity of intestinal junctions.

Specifically, we found elevated neurological antibodies with human subjects that had elevated levels of tight junction proteins in addition to statistically significant correlations between tight junction antibodies and neurological antibodies (Figure 2 and Table 2). During their breakdown, tight junction proteins in the intestinal barrier also have similar relationships with tight junction proteins in the blood–brain barrier, and the dysfunction of tight junction proteins has been theorized to play a role in neuroinflammatory conditions. Tight junction proteins have been found to play a role in immunological responses in the brain and are associated with elevations in the surrogate markers of blood–brain barrier permeability [10,11,12,13,14]. Patients suffering from relapsing–remitting multiple sclerosis were found to have a relatively high proportion of intestinal permeability compromise when compared to matched controls, suggesting that disturbance in the integrity of microbiota may play a role in the pathophysiology of multiple sclerosis. Intestinal permeability has been found to be a potential target site for the therapeutic treatment of multiple sclerosis, and the disruption of the intestinal tight junctions could lead to the early detection of autoimmune encephalomyelitis in animal models [15]. Our data support these previous studies and suggest a specific list of neurological autoimmune target proteins antibodies that may have a relationship with intestinal permeability.

We identified numerous autoimmune target antibodies to joint and bone sites that are correlated and elevated in subjects with elevated tight junction proteins (Figure 3 and Table 2). The integrity of the intestinal microbiota is found to be significantly different in patients with early rheumatoid arthritis [16,17]. Recent studies have suggested that intestinal permeability can lead to the circulation of arthritogenic bacteria and cause inflammation in synovial tissues [18,19,20]. Furthermore, inflammatory conditions in the bowel, involving the translocation of bacterial products against the endothelial gut barrier have been found to cause inflammation in the bone and impact what is known as the gut–microbiota–bone axis [21,22]. Our data provide further support that intestinal permeability may play a role in autoimmune and inflammatory reactions involving the protein target sites of both bone and synovial tissues.

The relationship between intestinal permeability and autoimmune diseases of the endocrine system has become an area of great interest for researchers. Several case–control studies have identified increased intestinal permeability in patients with type 1 diabetes compared to healthy controls [23,24,25,26]. Our study supports the findings of these previous studies and has determined that subjects with elevated tight junction antibody levels may have mean insulin/islet cell antibodies compared to those without elevated tight junction antibody levels. We also identified several other autoimmune antibody elevations that could be found with autoimmune thyroid disease (thyroglobulin Abs), Addison’s disease (21-hydroxylase Abs), and autoimmunity associated ovarian/testicular failure (ovary/tes-190 tis Abs) (Figure 4 and Table 2). The findings of our study suggest that intestinal permeability may have systemic autoimmune responses, involving a diverse list of autoimmune target proteins, and may potentially play a role in polyglandular autoimmune disease.

Our study may also indicate relationships between intestinal permeability/autoimmunity and gastrointestinal and hepatic autoimmune target proteins, such as parietal cell antibodies, ASCA antibodies, ANCA antibodies, cytochrome p450, and tropomyosin antibodies 196 (Figure 5 and Figure 6, and Table 2). These autoantibodies are found with primary sclerosing cholangitis, autoimmune hepatitis, gastric autoimmunity, and chronic inflammatory bowel diseases. Researchers have speculated that intestinal permeability may play a role in these specific autoantibodies and associated diseases [4,27,28,29].

## 4. Methods and Materials

### 4.1. Data Set

To study the relationship between intestinal permeability and autoimmunity in human subjects, the investigators contacted a clinical autoimmune specialty laboratory and requested a non-identifiable data set containing 200 or more human subjects that included ELISA (enzyme-linked immunosorbent assay) measurements of antibodies, both for intestinal permeability and antibodies as well as autoimmune target proteins. The laboratory is CLIA (Clinical Laboratory Improvement Amendments of 1988)-certified and monitored for proficient testing, compliance with laboratory guidelines, and standards of ELISA testing. The laboratory provided our investigation with a data set of 266 random human subjects containing the quantification of 24 different autoimmune target protein antibodies of the occludin/zonulin antibody (but not measures of occludin and zonulin antibodies separately). The collection of data was approved by the Institutional Review Board of Partners Healthcare at Massachusetts General Hospital. Age and gender data were provided but no further clinical or demographic data were made available.

Serum samples used in the data set were collected at multiple medical centers in the United States. Licensed physicians clinically selected subjects to be screened for intestinal permeability and autoimmunity based on the subject’s medical records and medical findings in each clinician’s files. The ages of subjects ranged between 19 and 87 years, with a mean age of 50. Sixty-five percent of the subjects were female and thirty-fix percent of the subjects were male. No further medical or demographic information was provided about the subjects to the autoimmune specialty laboratory or provided to us in the data set.

### 4.2. Statistical Analysis

Statistical analysis was performed using STATA software package 14.2. Logistic regression, Pearson’s correlation coefficients, and *t*-tests were conducted to analyze the data. Subjects with mean occludin/zonulin IgG antibodies that were two (2) or more standard deviations above the mean were classified as positive for intestinal permeability. This was carried out by converting continuous data from optical density measurements of zonulin/occludin antibodies to the binary classification of intestinal permeability as either “positive” or “negative”. Our analyses were conducted using a Bonferroni correction to adjust for a type I error with the significant *p*-value set to 0.002.

## 5. Conclusions

The worldwide prevalence of autoimmune diseases is rapidly rising with limited treatment options and preventive strategies [30]. There is growing evidence that the microbiota and the integrity of the intestinal barrier may play a role in autoimmune diseases [31]. Understanding these relationships could lead to medications, lifestyle modifications, nutraceuticals, and dietary strategies that may attenuate autoimmune expression [7]. These relationships may play a role in a spectrum of autoimmune diseases [32]. The potential to evaluate intestinal barrier integrity for susceptible individuals and to determine whether restoring intestinal junction integrity impacts autoimmune disease will be an important area of research in autoimmune disease development and future treatment strategies [33,34].

## Figures and Tables

**Table 1:** Odds ratio for developing elevated autoimmune target protein antibodies with occludin/zonulin positive subjects compared to the odds of developing barrier protein antibodies with occludin/zonulin negative subjects. The statistically significant *p*-value with a Bonferroni adjustment for multiple comparisons is <0.002.

Autoantibody	Odd Ratio	95% Confidence Interval	*p*-Value
Parietal Cell + ATPase IgG + IgA	5.8	2.6–12.8	<0.0001
Intrinsic Factor IgG + IgA	1.7	1.1–2.7	0.025
ASCA + ANCA IgG + IgA	8.4	3.5–20.2	<0.0001
Tropomyosin IgG + IgA	7.0	3.3–15.0	<0.0001
Thyroglobulin IgG + IgA	1.6	0.3–1.0	<0.034
Thyroid Peroxidase IgG + IgA	2.4	1.4–4.2	0.002
21 Hydroxylase IgG + IgA	28.0	8.7–89.0	<0.0001
Myocardial Peptide IgG + IgA	9.0	4.1–19.2	<0.0001
Alpha-Myosin IgG + IgA	10.4	4.5–23.1	<0.0001
Phospholipid IgG + IgA	2.5	1.3–5.0	0.009
Platelet Glycoprotein IgG + IgA	5.4	2.3–13.0	<0.0001
Ovary/Testis IgG + IgA	7.1	3.0–17.7	<0.0001
Fibulin IgG + IgA	7.2	3.0–17.0	<0.0001
Collagen Complex IgG + IgA	2.2	1.3–4.0	0.005
Arthritic Peptide IgG + IgA	30.0	10.0–77.8	<0.0001
Osteocyte IgG + IgA	6.6	2.8–15.7	<0.0001
Cytochrome P450 IgG + IgA	22.3	8.0–64.4	<0.0001
Insulin + Islet Cell Antibody IgG + IgA	0.9	0.6–1.6	<0.0001
Glutamic Acid Decarboxylase-65 IgG + IgA	10.0	4.2–23.3	<0.0001
Myelin Basic Protein IgG + IgA	6.9	3.2–15.0	<0.0001
Asialoganglioside IgG + IgA	6.2	2.9–13.3	<0.0001
Alpha + Beta Tubulin IgG + IgA	2.7	1.6–4.6	<0.0001
Cerebellar IgG + IgA	22.0	8.0–59.9	<0.0001
Synapsin IgG + IgA	8.7	3.3–22.5	<0.0001

**Table 2:** Correlation coefficients between zonulin/occludin IgG antibodies and autoimmune target protein antibodies. The statistically significant *p*-value with a Bonferroni adjustment for multiple comparisons is <0.002.

Autoantibody	Correlation Coefficient	*p*-Value
Parietal Cell + ATPase IgG + IgA	0.4	<0.0001
Intrinsic Factor IgG + IgA	0.3	<0.0001
ASCA + ANCA IgG + IgA	0.4	<0.0001
Tropomyosin IgG + IgA	0.4	<0.0001
Thyroglobulin IgG + IgA	0.2	0.02
Thyroid Peroxidase IgG + IgA	0.2	0.001
21 Hydroxylase IgG + IgA	0.4	<0.0001
Myocardial Peptide IgG + IgA	0.3	<0.0001
Alpha-Myosin IgG + IgA	0.5	<0.0001
Phospholipid IgG + IgA	0.3	<0.0001
Platelet Glycoprotein IgG + IgA	0.3	<0.0001
Ovary/Testis IgG + IgA	0.4	<0.0001
Fibulin IgG + IgA	0.3	<0.0001
Collagen Complex IgG + IgA	0.2	<0.0001
Arthritic Peptide IgG + IgA	0.2	0.0005
Osteocyte IgG + IgA	0.2	0.004
Cytochrome P450 IgG + IgA	0.3	<0.0001
Insulin + Islet Cell Antibody IgG + IgA	0.2	0.0004
Glutamic Acid Decarboxylase-65 IgG + IgA	0.4	<0.0000
Myelin Basic Protein IgG + IgA	0.3	<0.0000
Asialoganglioside IgG + IgA	0.3	<0.0001
Alpha + Beta Tubulin IgG + IgA	0.3	<0.0001
Cerebellar IgG + IgA	0.4	<0.0001
Synapsin IgG + IgA	0.3	<0.0001

## Data Availability

The data presented in this study are available on request from the corresponding author.
